# Superior Capsular Reconstruction Using an Acellular Dermal Xenograft or Allograft Improves Shoulder Function but Is Associated with a High Graft Failure Rate

**DOI:** 10.3390/jcm13164646

**Published:** 2024-08-08

**Authors:** Maximilian Hinz, Lorenz Fritsch, Hannes Degenhardt, Marco-Christopher Rupp, Lucca Lacheta, Lukas N. Muench, Andrea Achtnich, Sebastian Siebenlist, Bastian Scheiderer

**Affiliations:** Department of Sports Orthopaedics, Technical University of Munich, 81657 Munich, Germany

**Keywords:** shoulder, arthroscopy, rotator cuff tear, salvage procedure, SCR

## Abstract

**Objectives:** The purpose of the present study was to evaluate clinical and functional outcomes, graft integrity rate and progression of osteoarthritis after superior capsular reconstruction (SCR) at short-term follow-up. **Methods:** Consecutive patients that underwent SCR using an acellular dermal xeno- or allograft between May 2018 and June 2020 for the treatment of irreparable posterosuperior rotator cuff tears were included. Shoulder function (American Shoulder and Elbow Surgeons [ASES] score), pain (Visual Analog Scale [VAS] for pain) and active shoulder range of motion (ROM) were evaluated preoperatively and after a minimum of 24 months postoperatively. Isometric strength was measured at follow-up and compared to the contralateral side. Magnetic resonance imaging was performed to evaluate graft integrity and osteoarthritis progression (shoulder osteoarthritis severity [SOAS] score). **Results:** Twenty-two patients that underwent SCR using a xeno- (n = 9) or allograft (n = 13) were evaluated 33.1 ± 7.2 months postoperatively. Four patients in the xenograft group underwent revision surgery due to pain and range of motion limitations and were excluded from further analysis (revision rate: 18.2%). Shoulder function (ASES score: 41.6 ± 18.8 to 72.9 ± 18.6, *p* < 0.001), pain levels (VAS for pain: 5.8 ± 2.5 to 1.8 ± 2.0, *p* < 0.001) and active flexion (*p* < 0.001) as well as abduction ROM (*p* < 0.001) improved significantly from pre- to postoperatively. Active external rotation ROM did not improve significantly (*p* = 0.924). Isometric flexion (*p* < 0.001), abduction (*p* < 0.001) and external rotation strength (*p* = 0.015) were significantly lower in the operated shoulder compared to the non-operated shoulder. Ten shoulders demonstrated a graft tear at the glenoid (n = 8, 44.4%) or humerus (n = 2, 11.1%). Graft lysis was observed in seven shoulders (38.9%). The graft was intact in one shoulder (5.6%), which was an allograft. A significant progression of shoulder osteoarthritis was observed at follow-up (SOAS score: 42.4 ± 10.1 to 54.6 ± 8.4, *p* < 0.001). **Conclusions:** At short-term follow-up, SCR using an acellular dermal xeno- or allograft resulted in improved shoulder function and pain with limitations in active external rotation ROM and isometric strength. Graft failure rates were high and osteoarthritis progressed significantly. **Level of Evidence:** Retrospective cohort study, Level III.

## 1. Introduction

Superior capsular reconstruction (SCR) was described as a joint-preserving procedure for the treatment of irreparable posterosuperior rotator cuff tears [1]. The purpose of this technique is to improve shoulder function by (re-)centering the humeral head through restoration of superior joint stability [1,2,3]. Previous clinical studies have reported improvements in shoulder function and pain following SCR [4,5,6] that may be superior to other non-arthroplasty options, such as latissimus dorsi tendon transfer [7,8,9] or lower trapezius tendon transfer [10]. Further, outcomes were comparable for SCR and reverse total shoulder arthroplasty when performed in patients younger than 70 years old with SCR having the advantage of preserving the joint [11]. Superior capsular reconstruction has, however, also been associated with high complication and graft failure rates [5,6,12] and is technically challenging [13].

Therefore, efforts have been made to improve outcomes and decrease complication rates following SCR. In addition to refining the indication for surgery [14,15], this includes advancing the technical properties of this procedure, such as investigating different graft materials [16,17] and thicknesses [17,18,19], the concept of augmentation [20] and side-to-side suturing [21].

Although graft integrity is generally sought after, this may not be necessary for good functional outcomes [22,23]. Nevertheless, graft failure may be associated with superior migration of the humeral head and osteoarthritis progression.

The purpose of the present study was to evaluate clinical and functional outcomes, graft integrity rate and progression of osteoarthritis after SCR at short-term follow-up. It was hypothesized that improvements in clinical and functional outcomes, a low rate of graft failure and no significant progression of osteoarthritis would be observed.

## 2. Material and Methods

The present study was approved by the institutional review board of the Technical University of Munich (reference: 278/20 S) and conducted according to the Declaration of Helsinki. All patients gave their written and informed consent. Consecutive patients that underwent SCR with an acellular dermal xeno- or allograft between May 2018 and June 2020 were eligible for participation. Further inclusion criteria were as follows: the presence of a symptomatic irreparable supraspinatus tendon tear with or without a concomitant infraspinatus tendon tear and a minimum follow-up of 24 months. Extensive cuff tear arthropathy (Hamada grade ≥ 3) [24] or irreparable subscapularis tendon tear were defined as exclusion criteria.

### 2.1. Surgical Technique

Surgery was performed by the senior author in all patients (B.S.). Acellular dermal porcine xenografts (DX Reinforcement Matrix, Arthrex Inc., Naples, FL, USA) or acellular dermal allografts (Epiflex^®^, DIZG, Berlin, Germany) were used for reconstruction in all cases. Xenografts were used until March 2019 and allografts were used from April 2019 onwards.

**Figure 1:**
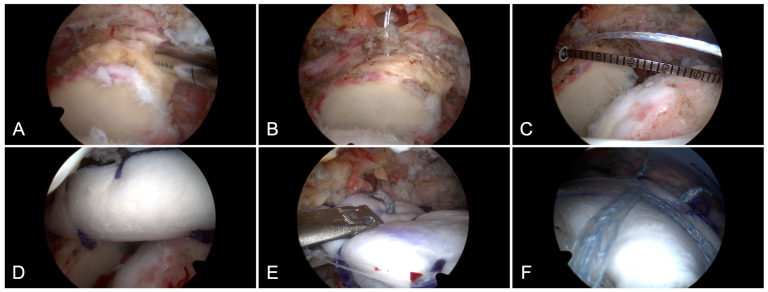
Arthroscopic superior capsular reconstruction. Debridement of the superior glenoid (**A**). Insertion of an all-suture anchor on the glenoid side at the 12-o’clock-position (**B**) and measurement of the defect size (**C**). Graft passage into the subacromial space (**D**). Graft fixation on the glenoid side (**E**). Graft fixation on the humeral side in a double-row configuration (**F**).

The surgical technique used has been described previously [25]. Patients were placed in the beach chair position. Following diagnostic arthroscopy, a standardized tenotomy of the long head of the biceps tendon was performed in all cases except revisions (in which the long head of the biceps tendon had previously been addressed surgically). Subacromial bursectomy and debridement of the superior glenoid and rotator cuff footprint were performed (Figure 1A). A single-loaded all-suture anchor (1.6 mm FiberTak^®^, Arthrex Inc., Naples, FL, USA) was inserted into the glenoid at the 12-o’clock position and 5 mm medial to the glenohumeral joint line (Figure 1B). The defect size was measured in anteroposterior and mediolateral direction using an arthroscopic measuring probe (Measurement Probe 70°, Arthrex Inc., Naples, FL, USA) in 30° of abduction and neutral rotation (Figure 1C). The graft was prepared according to the previous measurement with 10 mm added on the humeral side and 5 mm added each on the anterior, posterior and glenoid side. Then, the graft was folded into a double layer (xenograft, 3 mm thickness) or triple layer (allograft, 6 mm thickness) and secured using non-absorbable sutures (FiberWire #2, Arthrex Inc., Naples, FL, USA). Non-absorbable traction sutures were passed through the anterolateral and posterolateral corner of the graft, which were later used to adjust graft tension during the fixation on the humeral side. Using the suture limbs of the suture anchor at the 12-o’clock position, the graft was passed into the subacromial space (Figure 1D). The sutures were tied to achieve graft fixation. Two additional single-loaded all-suture anchors (1.6 mm FiberTak^®^, Arthrex Inc., Naples, FL, USA) were inserted at the 10- and 2-o’clock-position on the glenoid for fixation of the anteromedial and posteromedial corners of the graft (Figure 1E). Graft fixation on the humeral side was achieved in a double-row configuration (Figure 1F) using two suture anchors medially (4.75 mm SwiveLock^®^, Arthrex Inc., USA) and two suture anchors laterally (4.75 mm SwiveLock^®^, Arthrex Inc., USA). During this step, the non-absorbable sutures, which were inserted into the anterolateral and posterolateral corners of the graft earlier, were used to adjust graft tension. The graft was then sutured in a side-to-side configuration—posteriorly to the infraspinatus tendon and anteriorly to the rotator interval. Finally, proper graft fixation and tension were tested under full range of motion.

### 2.2. Postoperative Rehabilitation

The shoulder was immobilized in an abduction brace (medi SAS^®^ comfort, medi Bayreuth, Bayreuth, Germany) for 6 weeks. For the first three weeks, only passive mobilization was allowed with shoulder abduction and flexion limited to 45°. From the 7th postoperative week, patients progressed to active-assisted and subsequently active motion. Strengthening exercises were introduced from 12 weeks postoperatively.

### 2.3. Patient Characteristics and Operative Data

Chart and imaging review was performed to obtain demographic data, injury-related data and operative data. Fatty infiltration of the rotator cuff muscles was assessed on preoperative magnetic resonance imaging (MRI) using the adaptation of Fuchs et al. [26] of Goutallier’s classification and cuff tear arthropathy was assessed on preoperative radiographs using Hamada’s classification [24].

### 2.4. Clinical and Functional Outcome Assessment

Patient-reported outcome measures (PROMs) including American Shoulder and Elbow Surgeons (ASES) score [27], Subjective Shoulder Value (SSV) [28], Disabilities of the Arm, Shoulder and Hand (DASH) score [29], Constant-Murley score [30] and Visual Analog Scale (VAS) for pain as well as range of motion (ROM) were assessed pre- (ASES score, VAS for pain) and after a minimum of 2 years postoperatively (all PROMs and range of motion). At follow-up, isometric shoulder flexion strength (90° of shoulder flexion, neutral rotation), abduction strength (90° of shoulder abduction, neutral rotation), flexion strength and external rotation strength (0° of abduction, 90° of elbow flexion, neutral rotation) were measured unilaterally using an isometric dynamometer (ISOBEX, Cursor AG, Bern, Switzerland) and compared to the contralateral side. Complication and revision surgery rates were evaluated.

### 2.5. Magnetic Resonance Imaging

At final follow-up, patients underwent MRI of the shoulder (coronal: proton density-weighted, sagittal: T2-weighted, axial: proton density-weighted) to analyze graft integrity and osteoarthritis progression. The status of the graft was classified as intact, absent or torn (glenoid, mid-substance, humerus) according to Mirzayan et al. [31]. Further, MRI at follow-up was compared to preoperative MRI to evaluate the progression of osteoarthritis using the shoulder osteoarthritis severity (SOAS) score [32].

### 2.6. Subgroup Analyses

Postoperative shoulder function and osteoarthritis were compared between patients undergoing SCR using a xenograft vs. allograft SCR and between patients with glenoid-sided vs. humerus-sided graft tears to assess for a “biologic tuberoplasty effect” [23].

### 2.7. Statistical Analysis

Data were analyzed using SPSS 26.0 (IBM-SPSS, New York, USA). Categorical variables are presented in counts and percentages. Normal distribution of the collected continuous variables was assessed using the Shapiro–Wilk test and graphically confirmed. Normally distributed continuous variables are shown as mean ± standard deviation. Non-normally distributed continuous variables are shown as median (25–75% interquartile range). For group comparisons of continuous variables, the *t* test or Mann–Whitney U-test was applied. Statistical significance was set at a *p* value of <0.05. Furthermore, it was reported how many patients reached the minimally clinically important difference (MCID), substantial clinical benefit (SCB) and the patient-acceptable symptomatic state (PASS) for the ASES score, which were reported for SCR as 11.2, 18.02 and 68.82 in previous literature [33].

A post hoc power analysis was performed for the pre- to postoperative change in shoulder osteoarthritis (SOAS score) using two-tailed t tests to assess the statistical power of this study. It was shown that the included sample size could achieve an adequate power of 0.999 with an alpha of 0.05 (G*Power 3.1.9.6, Düsseldorf, Germany) [34].

## 3. Results

**Figure 2:**
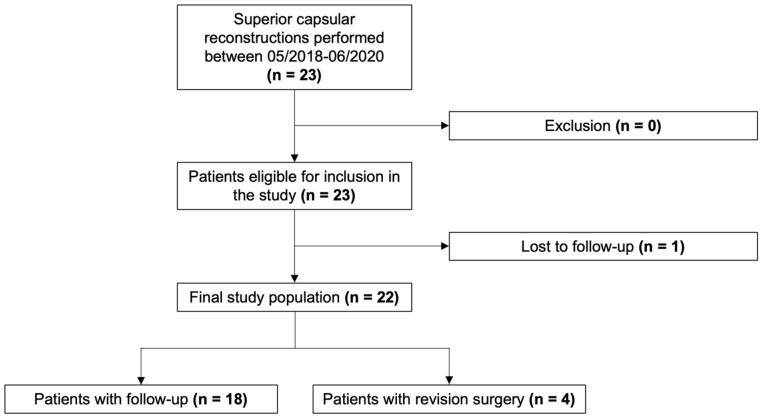
Flowchart for patient inclusion.

Superior capsular reconstruction was performed in 23 patients, of which 22 patients (95.7%) were included in the present study at a mean follow-up of 33.1 ± 7.2 months (Figure 2). A xenograft was used in nine patients (40.9%), and an allograft was used in 13 patients (59.1%). Four patients that underwent SCR with a xenograft underwent revision surgery due to pain and limited ROM, resulting in a revision rate of 18.2% (44.4% xenograft group, 0% allograft group). For revision procedures, two patients underwent reverse total shoulder arthroplasty (3 months postoperatively) and two patients underwent latissimus dorsi tendon transfer (8 and 9 months postoperatively) and were excluded from analyses. Detailed patient demographics and operative data are shown in Table 1. Preoperative fatty infiltration of the rotator cuff muscles and cuff tear arthropathy are reported in Table 2.

### 3.1. Clinical and Functional Outcome

Patient-reported outcome measures, ROM and strength measurements are reported in Table 3. Shoulder function, pain and active shoulder flexion as well as abduction ROM improved from preoperatively to final follow-up (*p* < 0.001). No improvement was observed for active external rotation ROM (*p* = 0.924). Isometric flexion (*p* < 0.001), abduction (*p* < 0.001) and external rotation strength (*p* = 0.015) were significantly lower in the operated shoulder compared to the non-operated shoulder. The majority of patients reached the MCID (n = 14, 77.8%) and SCB (n = 12, 66.7%), whereas only half of the patients achieved the PASS for the ASES score.

### 3.2. Radiological Outcome

**Figure 3:**
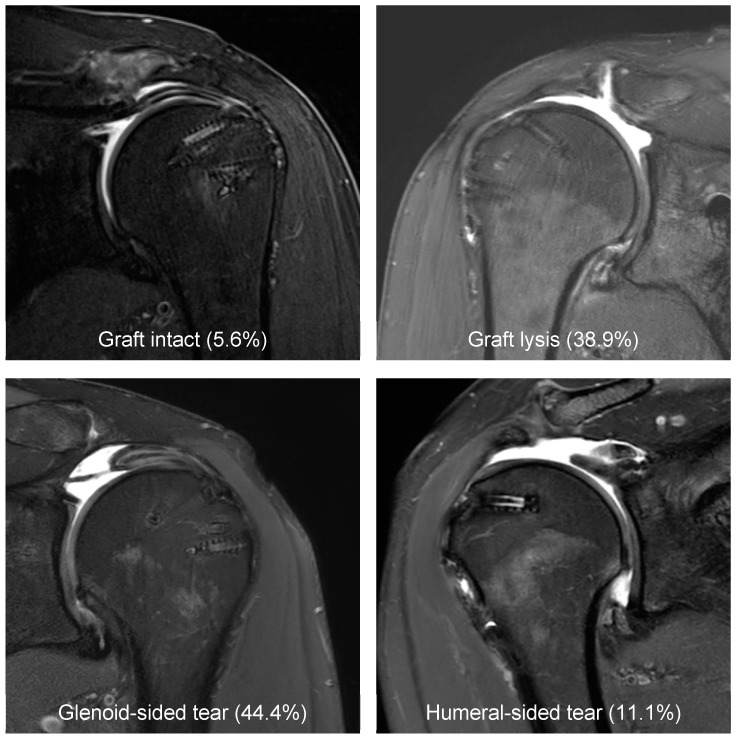
Graft integrity was evaluated with magnetic resonance imaging.

Ten shoulders demonstrated a graft tear either at the glenoid side (n = 8, 44.4%) or humeral side (n = 2, 11.1%). Graft lysis was observed in seven patients (38.9%). In one patient, the graft was intact (5.6%), which was an allograft (allograft healing rate: 7.7%; Figure 3). Mid-substance graft tears were not observed. A significant progression of osteoarthritis was observed from preoperatively to the final follow-up (SOAS score: 42.4 ± 10.1 vs. 54.6 ± 8.4, *p* < 0.001), respectively.

### 3.3. Subgroup Analyses

No significant differences were observed regarding improvement of shoulder function and overall postoperative shoulder function, progression of osteoarthritis or overall postoperative osteoarthritis between patients that underwent SCR with a xenograft or allograft (*p* > 0.05).

In case of graft failure, patients with glenoid-sided tears had a significantly greater reduction in pain (*p* = 0.003) and better postoperative shoulder function (Constant-Murley score: *p* = 0.021; SSV: *p* < 0.001), as well as significantly less severe shoulder osteoarthritis postoperatively, when compared with patients with humeral-sided graft tears (Table 4).

## 4. Discussion

The most important finding of the present study was that SCR using an acellular dermal xenograft or allograft resulted in a low graft healing rate and consequently, a significant progression of shoulder osteoarthritis at short-term follow-up. Nonetheless, a significant improvement of shoulder function was observed with 77.8% of patients reaching the MCID and half of patients reaching the PASS for the ASES score. Patients with a glenoid-sided graft tear, and thus a persistent coverage of the tuberosity, demonstrated a significantly greater reduction in pain and better postoperative shoulder function (SSV and Constant-Murley-Score) and less severe shoulder osteoarthritis at final follow-up compared to humeral-sided tears. Almost half of patients that underwent SCR with a xenograft (44.4%) underwent revision surgery by follow-up, whereas no patient with an allograft SCR underwent revision surgery.

Although shoulder pain, function and active ROM (flexion and abduction) improved, significant impairments of shoulder flexion, abduction and internal rotation strength were observed at follow-up when compared to the contralateral side. Considerable strength deficits were also observed in other studies following SCR [35,36] as well as after alternative treatment options for irreparable rotator cuff tears [37], including partial repair [38] or interposition grafting [38]. In a recent study by Marigi et al. [10], however, it was reported that SCR may lead to greater improvements of shoulder flexion strength rather than lower trapezius tendon transfer. Nonetheless, tendon transfers may restore the range of motion, specifically external rotation, to a greater degree than SCR [10,39].

The graft integrity rate in the present study was 5.6%, which correlates to a graft tear rate of 94.4%. Previous studies have reported considerably lower graft tear rates (7–83.3%) [4,12,40,41,42]. These differences may be in part related to the high proportion (77.8%) of revision cases and patients with fatty infiltration (Goutallier grade ≥ 2) of the infraspinatus muscle (94.4%) in the present study, both of which have been associated with inferior outcomes following SCR [43]. Graft options for SCR include, but are not limited to, acellular dermal xenografts [4,40], tensor fascia lata autografts [4], acellular dermal allografts [4,22,41], achilles tendon–bone allografts [12] and long head of the biceps tendon autografts [4,42]. Overall, xenograft use was associated with the highest graft tear (68%) [40] and reoperation rate (18–25%) [40,44]. Tensor fascia lata autografts, acellular dermal allografts and long head of the biceps tendon autografts showed the lowest graft tear rates (9%, 7% and 8%, respectively) and reoperation rates (3%, 6% and 9%, respectively) [4,42]. In the present study, the reoperation rate in the xenograft group was 44.4% and 0% in the allograft group. Beyond graft material, this difference may also be attributable to other factors, such as the learning curve associated with higher reoperation rates in earlier cases [45] and a smaller graft thickness due to a lower number of graft plies in the xenograft group [17,18,19]. Furthermore, it may be related to the difference in follow-up time as patients with allograft SCR had shorter follow-up time frames than those that underwent SCR using a xenograft. This effect is, however, thought to be limited as all patients in the xenograft group were revised upon in the first postoperative year. Graft tear rates, unlike reoperation rates, may be difficult to compare between studies. Whereas some studies, including the present study, defined a graft tear as a full-thickness tear on MRI, one study evaluated graft integrity via ultrasound, several studies defined graft tear as a failure to achieve the MCID and others reported no definition for graft tears [4,40]. Likewise, the indication for SCR, surgical technique used, postoperative immobilization and follow-up time frames varied between studies [4]. In the past, SCR has also been performed in patients with an irreparable subscapularis tear in combination with an irreparable posterosuperior rotator cuff tear. As recent studies reported that SCR may be associated with significantly inferior functional outcomes and higher graft tear rates in patients with irreparable subscapularis tears [14,15], the indication for SCR has since been adapted to exclude those patients.

In the present study, patients with glenoid-sided graft tears had a significantly greater reduction in pain, better postoperative shoulder function and less severe shoulder osteoarthritis at follow-up than patients with humeral-sided graft tears. Non-inferior outcomes for torn grafts or glenoid-sided graft tears (with a covered tuberosity) were also reported in previous studies [22,23]. This may be related to the graft’s ability to act as an interpositional spacer—especially in patients with a covered tuberosity—exhibiting a “biologic tuberoplasty effect” [23]. Nonetheless, shoulder osteoarthritis progressed in almost all patients, including in the one patient with an intact SCR graft, indicating that SCR is unable to prevent the progression of shoulder osteoarthritis. Osteoarthritis may, however, progress to a greater extent in patients with graft tears compared to patients with intact grafts [46].

There are some limitations present within the present study. First, the overall number of patients was low, which may be related to the narrow indication for SCR. Second, outcomes beyond failure rate may be difficult to compare between the xenograft vs. allograft group due to the difference in follow-up time and the high revision rate in the xenograft group. Third, the effect of graft integrity on the prevention of osteoarthritis progression could not be analyzed sufficiently, as only one patient had an intact graft at follow-up.

## 5. Conclusions

At short-term follow-up, SCR using an acellular dermal xeno- or allograft resulted in improved shoulder function and pain. Yet, graft failure rates were high and osteoarthritis progressed significantly.

## Figures and Tables

**Table 1:** Patient demographics and operative data.

	Mean ± SD or No. (%)
Total patient cohort	18
Age at time of surgery (years)	54.0 ^a^ (47.8–58.3)
Sex (male/female)	12/6 (66.7% male)
Surgery performed on the dominant side	11 (61.1%)
BMI (kg/m²)	26.8 ± 4.0
Tobacco use (%)	11 (55.6%)
Prior ipsilateral shoulder surgery	14 (77.8%)
Patients with concomitant diseases Rheumatoid arthritis (shoulder not affected)	1 (5.6%)
Concomitant procedures Long head of the biceps tendon tenotomy Infraspinatus tendon refixation	2 (11.1%) 2 (11.1%)

Normally distributed continuous variables are presented as mean ± standard deviation. Non-normally distributed continuous variables are presented as median (25–75% interquartile range). Categorical variables are presented as count (percentage). ^a^ Values are median.

**Table 2:** Preoperative grading of fatty infiltration of the rotator cuff muscles (Goutallier’s classification) and cuff tear arthropathy (Hamada’s classification).

	0	1	2	3	4
**Fatty infiltration**					
Supraspinatus	0	0	1 (5.6%)	10 (55.6%)	7 (38.9%)
Infraspinatus	0	1 (5.6%)	1 (5.6%)	10 (55.6%)	6 (33.3%)
Subscapularis	0	16 (88.9%)	2 (11.1%)	0	0
Teres minor	0	15 (83.3%)	2 (11.1%)	1 (5.6%)	0
	0	1		2	
**Cuff tear arthropathy**	0	12 (66.7%)		6 (33.3%)	

Categorical variables are presented as count (percentage).

**Table 3:** Patient-reported outcome measures, range of motion and postoperative strength measurements.

Outcome Parameter	Preoperative	Follow-Up	*p* Value
ASES score	41.6 ± 18.8	72.9 ± 18.6	**<0.001**
DASH score		28.7 ± 18.8	n.a.
Constant-Murley score		54.8 ± 16.3	n.a.
VAS for pain	5.8 ± 2.5	1.8 ± 2.0	**<0.001**
Active range of motion (°) Flexion Abduction External rotation	90.0 ^a^ (60.0–155.0) 80.0 ^a^ (45.0–112.5) 30.0 ^a^ (17.5–45.0)	150.0 ^a^ (95.0–170.0) 142.5 ^a^ (105.0–162.5) 30.0 ^a^ (20.0–42.5)	<**0.001** <**0.001** 0.924
	**Operated shoulder**	**Non-operated shoulder**	
SSV at follow-up	64.4 ± 22.3	87.8 ± 17.6	**0.002**
Isometric strength at follow-up (kg) Flexion Abduction External rotation	1.9 ^a^ (1.5–2.5) 1.5 ^a^ (1.0–2.3) 3.6 ^a^ (2.5–5.7)	4.9 ^a^ (3.1–8.5) 5.0 ^a^ (2.8–6.9) 6.1 ^a^ (4.3–8.3)	**<0.001** **<0.001** **0.015**

Normally distributed continuous variables are shown as mean ± standard deviation. Non-normally distributed continuous variables are shown as median (25–75% interquartile range). ASES American Shoulder and Elbow Surgeons, DASH Disabilities of the Arm, Shoulder and Hand, SSV Subjective Shoulder Value, VAS Visual Analog Scale. Bolded *p* values indicate statistical significance. ^a^ Values are median.

**Table 4:** Difference in radiological and functional outcomes between patients with humeral-sided vs. glenoid-sided SCR graft tears.

Outcome Parameter	Humeral-Sided Graft Tear (*n* = 2)	Glenoid-Sided Graft Tear (*n* = 8)	*p* Value
Preoperative VAS for pain	4.5 ± 3.5	6.0 ± 2.2	0.655
Postoperative VAS for pain	3.5 ± 3.5	1.3 ± 1.5	0.530
∆VAS for pain pre- to postoperative	−1.0 ± 0	−4.8 ± 2.4	**0.003**
Preoperative ASES score	40.0 ± 17.0	42.5 ± 19.8	0.876
Postoperative ASES score	55.0 ± 24.0	76.5 ± 17.1	0.411
∆ASES score pre- to postoperative	15.0 ± 7.1	34.0 ± 30.5	0.150
DASH score	47.9 ± 24.2	28.3 ± 19.3	0.442
Constant-Murley score	37.5 ± 2.1	54.9 ± 16.4	**0.021**
SSV	30.0 ± 0	70.0 ± 17.7	**<0.001**
Preoperative SOAS score	56.5 ± 10.6	40.3 ± 8.9	0.235
Postoperative SOAS score	64.0 ± 2.8	50.3 ± 10.3	**0.017**
∆SOAS score pre- to postoperative	7.5 ± 7.8	10.0 ± 8.2	0.736

Normally distributed continuous variables are shown as mean ± standard deviation. Non-normally distributed continuous variables are shown as median (25–75% interquartile range). ASES American Shoulder and Elbow Surgeons, DASH Disabilities of the Arm, Shoulder and Hand, SCR superior capsular reconstruction, SOAS shoulder osteoarthritis severity, SSV Subjective Shoulder Value, VAS Visual Analog Scale. Bolded *p* values indicate statistical significance.

## Data Availability

Due to ethical requirements, the data presented in this study are available from the corresponding author only upon request.
